# Suppressive Role of PPARγ-Regulated Endothelial Nitric Oxide Synthase in Adipocyte Lipolysis

**DOI:** 10.1371/journal.pone.0136597

**Published:** 2015-08-28

**Authors:** Yoko Yamada, Masato Eto, Yuki Ito, Satoru Mochizuki, Bo-Kyung Son, Sumito Ogawa, Katsuya Iijima, Masao Kaneki, Koichi Kozaki, Kenji Toba, Masahiro Akishita, Yasuyoshi Ouchi

**Affiliations:** 1 Department of Geriatric Medicine, Graduate School of Medicine, University of Tokyo, Tokyo, Japan; 2 Department of Anesthesia, Critical Care and Pain Medicine, Massachusetts General Hospital, Shriners Hospitals for Children, Harvard Medical School, Boston, Massachusetts, United States of America; 3 Department of Geriatric Medicine, Kyorin University School of Medicine, Tokyo, Japan; INRA, FRANCE

## Abstract

**Introduction:**

Metabolic syndrome causes insulin resistance and is associated with risk factor clustering, thereby increasing the risk of atherosclerosis. Recently, endothelial nitric oxide synthase deficient (eNOS-/-) mice have been reported to show metabolic disorders. Interestingly, eNOS has also been reported to be expressed in non-endothelial cells including adipocytes, but the functions of eNOS in adipocytes remain unclear.

**Methods and Results:**

The eNOS expression was induced with adipocyte differentiation and inhibition of eNOS/NO enhanced lipolysis in vitro and in vivo. Furthermore, the administration of a high fat diet (HFD) was able to induce non-alcoholic steatohepatitis (NASH) in eNOS-/- mice but not in wild type mice. A PPAR**γ** antagonist increased eNOS expression in adipocytes and suppressed HFD-induced fatty liver changes.

**Conclusions:**

eNOS-/- mice induce NASH development, and these findings provide new insights into the therapeutic approach for fatty liver disease and related disorders.

## Introduction

Metabolic syndrome is well known to contribute to the increase in risk of atherosclerotic cardiovascular disease [1]. Excess energy expenditure results in spillover of energy storage from adipose tissue to the liver, and fatty liver is known to be more strongly related to insulin resistance and hypertriglyceridemia than visceral adipose tissue [2]. Recently, adipose tissue has been shown to be not only a passive energy reservoir but also one of the largest endocrine organs in the body, secreting various types of humoral factors that are collectively called adipokines. Among such adipokines, free fatty acids (FFAs) cause lipotoxicity and lead to insulin resistance as a result of the non-oxidative metabolism of its ectopic deposits in visceral organs such as the liver [3]. Physiologically, in response to the energy demand of peripheral tissues, catecholaminergic stimulation of the β-adrenergic receptor triggers the process of lipolysis whereby accumulated triglycerides in intracellular lipid droplets of adipocytes are broken down into FFAs and glycerol.

eNOS generates nitric oxide (NO) from L-arginine in endothelial cells and plays a pivotal role in blood flow regulation and vascular homeostasis [4]. Consistent with this function of eNOS, recent studies have shown that eNOS^-/-^ mice exhibit a hypertensive phenotype as anticipated, but surprisingly they also had disorders that are hallmarks of the metabolic syndrome, namely insulin resistance, dyslipidemia and obesity [5, 6]. In this regard, a previous report has indicated that the pathological mechanism of insulin resistance in eNOS^-/-^ mice was related to defective skeletal muscle glucose uptake [7]. On the other hand, it has also been shown that in addition to endothelial cells, eNOS is heterotopically expressed in several other types of cells including adipocytes [5, 6, 8], and deficiency of heterotopic eNOS might contribute to the metabolic phenotype and insulin resistance observed in these deficient mice. However, the roles of heterotopic eNOS in this context remain unclear.

The purpose of this study was to determine the roles of eNOS in adipocytes. We found that adipocyte eNOS has an antilipolytic action and eNOS was associated with non-alcoholic steatohepatitis (NASH) formation. We also showed a role for PPARγ in negatively regulating eNOS expression in adipocytes, which has important therapeutic implications for the treatment of fatty liver and related disorders.

## Methods

### Animals

C57BL/6J mice and eNOS^-/-^ mice (4–8 weeks of age) were obtained from Charles River Laboratories Japan. Mice were housed in a specific pathogen-free barrier facility at 22±2°C with a 12-hour light/dark cycle and relative humidity of 40–60%, with free access to water and a standard chow diet (53% carbohydrate, 6% fat, 25% protein calories). In some experiments, mice were fed a HFD (D12492; 26% carbohydrate, 35% fat, 26% protein calories) obtained from LSG Corporation. GW9662 (10 mg/kg) was administered intraperitoneally on alternate days for 19 days. All animal experiments were approved by the University of Tokyo Ethics Committee for Animal Experiments (approval number: M-P-10-032) and strictly adhered to the guidelines for animal experiments of the University of Tokyo.

### Metabolic Measurements

Mice were fasted overnight (for 16 hour) and then blood was collected. Enzymatic assay kits were used for determination of cholesterol (Determiner TC; Kyowa Medex), TG (Determiner L TG II; Kyowa Medex), and FFAs (Wako NEFA C test kit; Wako Chemicals), and ALT (transaminase C- II: Wako Pure Chemicals). Serum glucose was measured with a glucose analyzer (Bayer Medical). Serum insulin was determined with a mouse insulin enzyme-linked immunosorbent assay kit (Wako Chemicals). For ITTs, mice in a randomly fed state were injected intraperitoneally with human regular insulin (0.75 U/kg: Eli Lilly). Blood was collected before injection and at different times after injection, and glucose and insulin values were determined.

### Histological Examination

Fresh tissues (liver and fat) were collected and fixed in 10% neutral buffered formalin solution, embedded in paraffin, and 8-μm-thick sections were cut from the paraffin blocks for staining with hematoxylin and eosin, and Sirius red.

### Immunohistochemical Study

Paraffin-embedded serial sections of liver tissue were stained immunohistochemically with anti-CD68 (PG-M1, DAKO) or anti- 8-hydroxydeoxyguanosine (Chemicon) antibodies. Briefly, after deparaffinization and rehydration, the antigen retrieval procedure was applied by heating the slides in 0.1 M citrate buffer (pH 6.0) for 10 minutes. The sections were then incubated first with 3% H_2_O_2_ in distilled water for 5 minutes to block endogenous peroxidase and then incubated with monoclonal mouse anti-CD68 for 30 minutes or polyclonal goat anti-8-OHdG (10 mg/mL) at 4°C overnight, followed by biotinylated secondary antibody conjugated with avidin-biotin horseradish peroxidase (Dako Envison kit/HRP; Dako), and after several washes the slides were counterstained with 4^’^,6-diamidino-2-phenylindole.

### Liver Lipid Test

Liver tissues were homogenized in PBS (1 g: 20 ml). Lipids were extracted from liver tissue lysate using a chloroform/methanol (1:2) mixture. TG and glycerol were determined using a Determiner L TG II kit (Kyowa Medex, Japan).

### Cell Culture

The 3T3L1 preadipocytic cell line was purchased from Health Science Research Resources Bank and maintained in Dulbecco’s modified Eagle medium (DMEM) containing 25 mM HEPES buffer, penicillin (Sigma Aldrich), and 10% fetal FBS. Cells were grown to confluence in 7.5 cm flasks and treated with differentiation medium at 2 days post-confluence at 37°C in a humidified atmosphere with 5% CO_2_. For differentiation studies, basal DMEM medium was supplemented with 10% FBS and a differentiation cocktail composed of 1 μg/mL insulin, 0.5 mM IBMX, and 0.25 μM dexamethasone. Two days post-induction of differentiation, the cells were maintained in medium supplemented with 0.5 μg/mL insulin. Medium supplemented with 10% FBS was used to culture undifferentiated cells.

### Materials

3-Isobutyl-1-methyl-xanthine (IBMX), insulin, dexamethasone, and isoproterenol were purchased from Sigma Chemical Co. l-N5-(1-lminoethyl) ornithine dihydrochloride (L-NIO), KT5720, ODQ, wortmannin, and diethylamine NONOate (DeaNONOate) were from CALBIOCHEM, troglitazone, GW9662, and ciglitazone were from CAYMAN CHEMICAL COMPANY, and auranofin was from Alexis Biochemicals.

### Measurement of Lipolysis

Mature 3T3L1 adipocytes were preincubated with L-NAME, L-NIO, ODQ, vardenafil for 1 hour, DeaNONOate for 10 min, auranofin, DNCB for 2 hours, or with eNOS-siRNA for 48 hours. The cells were then washed twice with PBS and lipolysis was induced by the addition of isoproterenol (10 μM) or vehicle for 1 hour. Glycerol level in the incubation medium was used as an index of lipolysis and measured by a colorimetric method using the Adipolysis assay kit (Chemicon). The results were corrected against the basal level and expressed as relative units.

### Detection of S-nitrosylation in Cells Using Biotin Switch Method

Expression of S-nitrosylated proteins in cells was detected by the biotin switch method using an S-Nitrosylated Protein Detection Assay Kit (CAYMAN CHEMICAL COMPANY) in combination with fluorophore labeling and visualization by confocal microscopy [9].

### Luciferase Assay

3T3L1 preadipocytes were pretreated with vehicle or ciglitazone (10μmol/L) with insulin, dexamethasone and IBMX for 48 hours, then cells were seeded in 12-well plates at a density of 2.5×10^4^ cells/well and transiently transfected with 0.8μg of human pGL2-eNOS promoter-luciferase plasmid construct (Addgene) using Lipofectamine LTX & Plus Reagent (Invitrogen) according to the procedure recommended by the manufacturer. After 24 hours, 20 μL aliquots of cleared lysate were assayed with a luciferase assay kit from Promega.

### Statistical Analysis

All results are presented as mean ±SEM. Statistical comparisons were made by ANOVA, followed by Bonferroni test. A value of p < 0.05 was considered to be statistically significant.

## Results

### Induction of eNOS Expression in Differentiating Adipocytes

**Fig 1:**
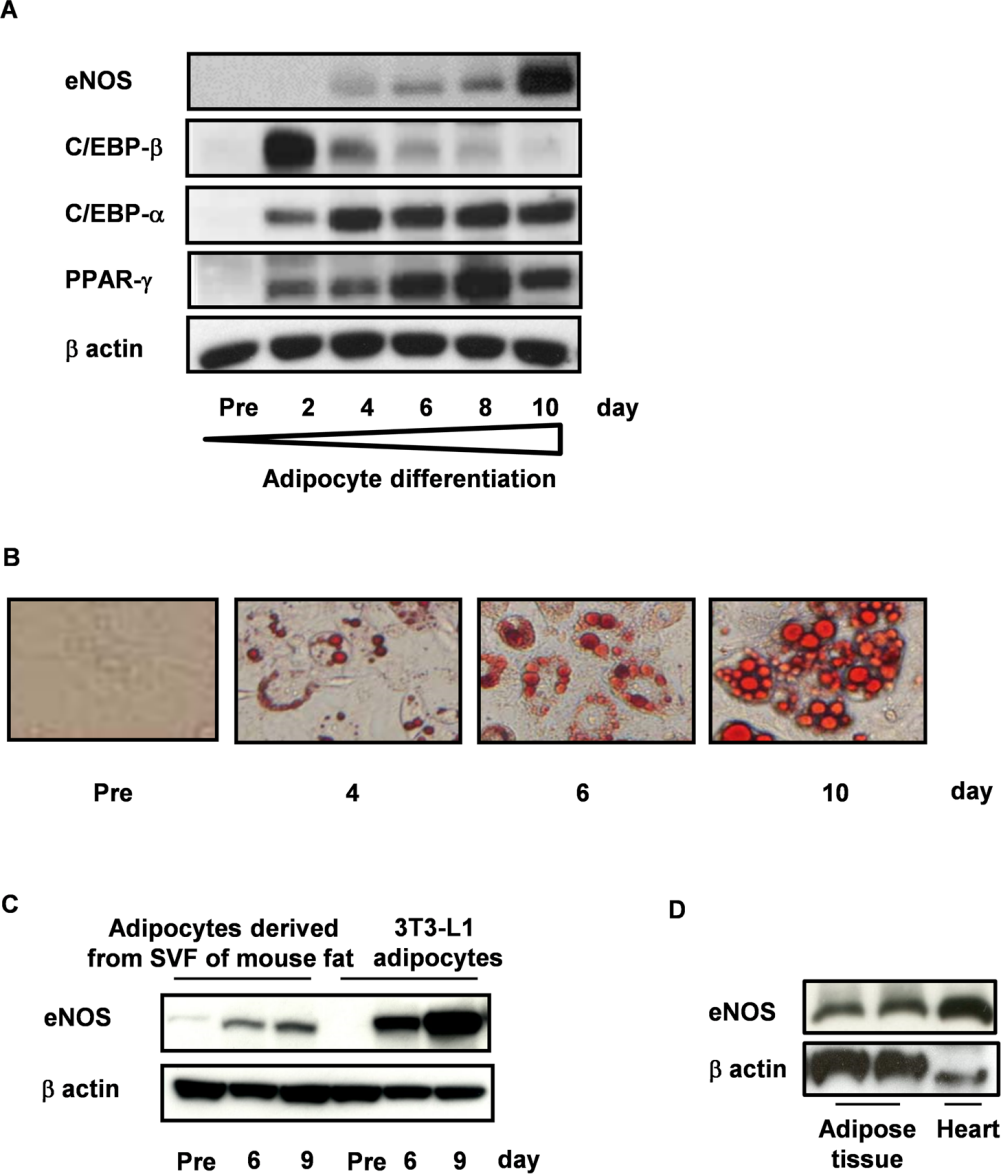
Expression of eNOS in differentiating adipocytes. (A) Protein expression during 3T3L1 adipocyte differentiation was examined by immunoblot analysis. (B) Oil red O staining during 3T3L1 differentiation. Photos were taken under a microscope with a ×20 objective lens. (C) SVF from the adipose tissue of HFD-fed wild type (WT) mice or 3T3L1 preadipocytes were stimulated with insulin, IBMX and dexamethasone to differentiate into mature adipocytes, and then analyzed by immunoblot analysis with an antibody against eNOS. (D) Extracts prepared from the epididymal fat and hearts (positive control) of WT mice were examined by immunoblot analysis with an antibody against eNOS.

As shown in Fig 1A, eNOS expression at both the protein and mRNA level was markedly induced during the differentiation of adipocytes while it was essentially undetectable in preadipocytes (Fig 1A and 1B and S1A and S1B Fig). Consistent with this finding, NO signal was also detected in mature adipocytes (S1C Fig). In contrast, neither iNOS nor nNOS mRNA was detected in either preadipocytes or differentiating adipocytes (S1A Fig). A similar pattern of eNOS expression was observed using primary cultured (pre) adipocytes derived from the stromal-vascular fraction (SVF) of mouse adipose tissue (Fig 1C). Furthermore, eNOS was strongly detected in the epididymal adipose tissue of wild type (WT) mice (Fig 1D), where the expression was mainly detected in the mature adipocyte fraction rather than in the SVF (S1D Fig).

### eNOS in Adipocytes Possess an Anti-Lipolytic Action

To clarify the functional role of eNOS in adipocytes, we first investigated its effect on adipocyte differentiation. However, use of L-NIO (a specific eNOS inhibitor) and siRNA-mediated knockdown of eNOS in 3T3L1 adipocytes had no effect on its differentiation (S2A–S2C Fig).

**Fig 2:**
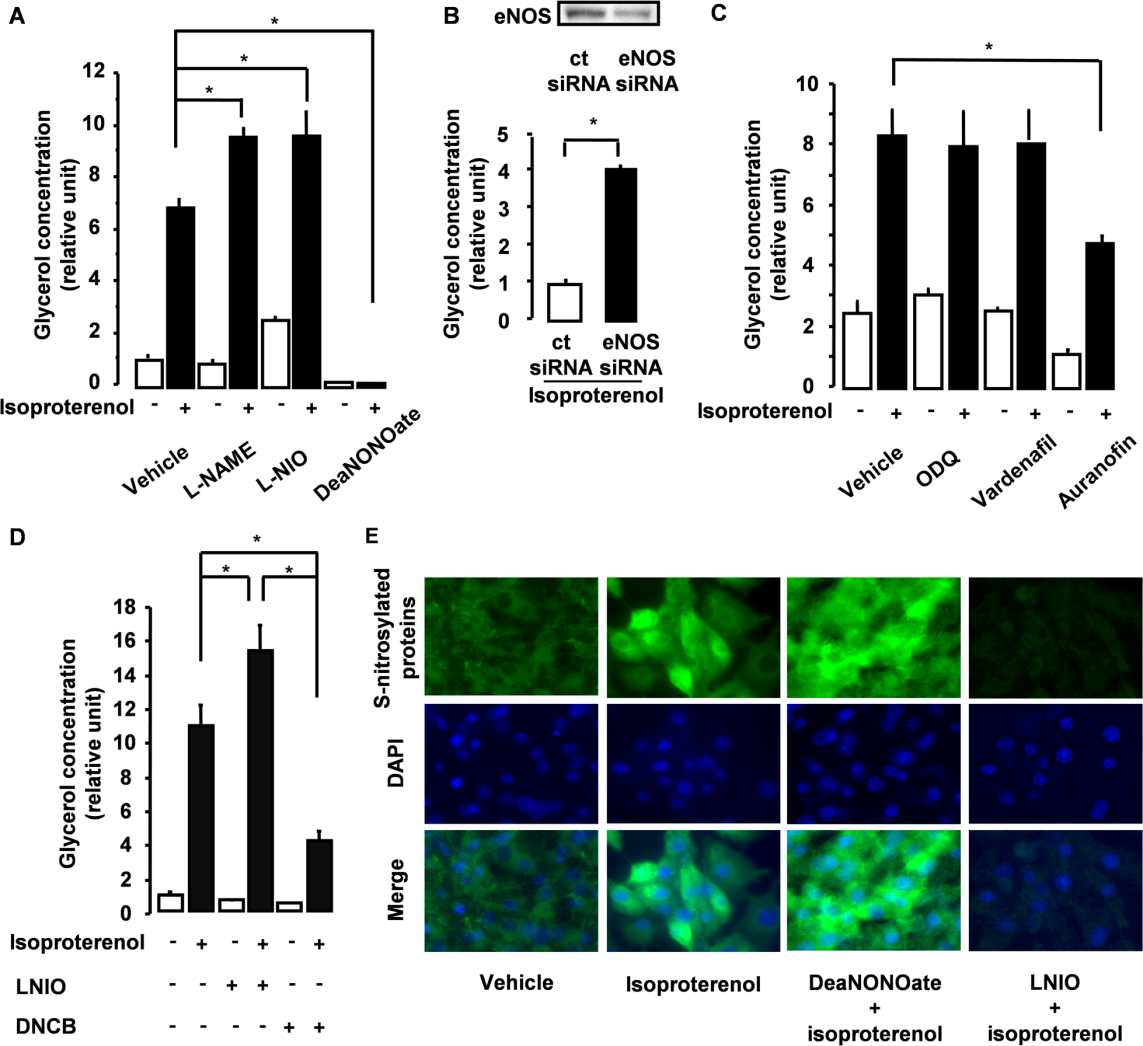
Adipocyte-specific eNOS has a suppressive effect on lipolysis. (A-D) After pretreatment, lipolysis was induced by the addition of vehicle or isoproterenol (10 μM) to mature 3T3L1 adipocytes (day 10) (n = 3–6). (A) Adipocytes were pretreated with vehicle, L-NAME (1 mM), L-NIO (10 μM), or DeaNONOate (10 mM). (B) Adipocytes were pretreated with control siRNA or eNOS-siRNA (200 pM), and examined by immunoblot analysis with an antibody against eNOS. (C) Adipocytes were pretreated with vehicle, ODQ (10 μM), vardenafil (10 μM), or auranofin (2 μM). (D) Adipocytes were pretreated with vehicle, L-NIO, or DNCB (10 μM). *p < 0.05. All values are expressed as mean ±SEM. (E) Adipocytes were pretreated with vehicle, isoproterenol, DeaNONOate + isoproterenol, or L-NIO + isoproterenol. The cells were then fixed and subjected to biotin derivatization, incubated with streptavidin-fluorescein isothiocyanate, and photos were taken under confocal microscopy with a ×40 objective lens.

We next focused on lipolysis, one of the most important processes in the adipocyte as it provides the means for releasing FFAs from adipocytes. When energy is needed, catecholaminergic stimulation of the β-adrenoreceptor leads to the breakdown of accumulated triglycerides in adipocyte intracellular lipid droplets into FFAs and glycerol. Thus, we performed a lipolysis assay to determine whether eNOS in adipocytes could affect lipolysis. β-adrenergic receptor stimulation of 3T3L1 adipocytes by isoproterenol markedly induced lipolysis (Fig 2A) that was associated with activation of the hormone sensitive lipase (HSL) pathway (S2D Fig), consistent with the results of previous studies. Isoproterenol also induced phosphorylation of both Akt and eNOS (S2D Fig). Meanwhile, pretreatment of 3T3L1 adipocytes with wortmannin (a PI3K inhibitor) suppressed eNOS phosphorylation (S2E Fig), indicating that eNOS is activated by isoproterenol through the PI3K/Akt pathway. Under these conditions, mature 3T3L1 adipocytes were pretreated with L-NAME (a NOS inhibitor), L-NIO, eNOS-targeting siRNA, or DeaNONOate (a NO donor) to examine the role of eNOS in lipolysis. Inhibition of eNOS in adipocytes significantly augmented lipolysis whereas DeaNONOate significantly suppressed lipolysis (Fig 2A and 2B). One of the mechanisms underlying the cellular effects of NO is through the activation of soluble guanylate cyclase (sGC), which leads to increased intracellular cGMP. However, ODQ (an inhibitor of sGC) and vardenafil (an inhibitor of PDE5) had no significant effect on lipolysis (Fig 2C). Besides the cGMP pathway, functional regulation of target proteins by post-translational modification (S-nitrosylation) has also been reported to play a role in the biological function of NO [10, 11]. Indeed, auranofin and DNCB (both thioredoxin reductase inhibitors that promote S-nitrosylation) significantly suppressed lipolysis, a result that is consistent with the involvement of S-nitrosylation in this process (Fig 2C and 2D). Furthermore, S-nitrosylation was promoted by DeaNONOate and suppressed by L-NIO, suggesting that the antilipolytic effect of eNOS/NO is mediated by S-nitrosylated proteins in adipocytes (Fig 2E).

### Excessive Lipolysis of eNOS^-/-^ Mice Fed a HFD

**Fig 3:**
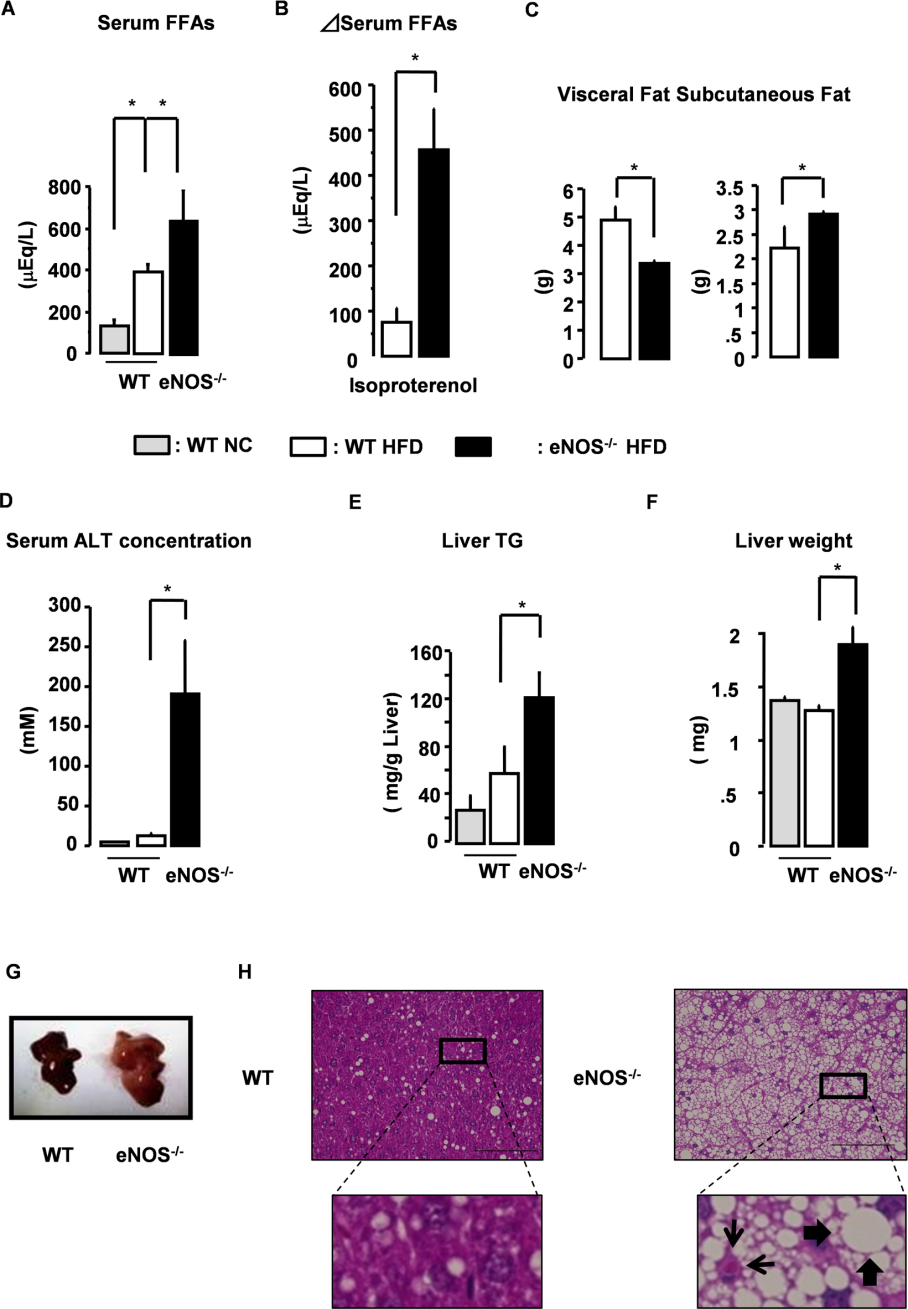
Hepatic steatosis in eNOS^-/-^ mice on HFD. (A) Serum FFA level in NC- and HFD-fed WT and eNOS^-/-^ mice (n = 4–8). *p < 0.05. All values are expressed as mean ±SEM. (B) HFD-fed WT and eNOS^-/-^ mice (n = 5) were injected with isoproterenol (10 mg/kg) interperitoneally. Bars show the difference in serum FFA levels at 6 hours after injection compared to before the injection (n = 5–7). (C) Weight of visceral fat and subcutaneous fat in HFD-fed WT and eNOS^-/-^ mice. Data represent mean ± SE (n = 5–6). Statistically significant differences are indicated (*p < 0.05). (D) Serum ALT level, (E) liver TG, and (F) liver weight in NC- and HFD-fed WT and eNOS^-/-^ mice (n = 4–8). *p < 0.05. All values are expressed as mean ±SEM. (G) Livers of HFD-fed WT and eNOS^-/-^ mice. (H) H&E-stained livers of HFD-fed WT and eNOS^-/-^ mice. Thick arrow indicates ballooning of hepatocytes. Thin arrow indicates Mallory body. Scale bar, 100 μm.

Next, to determine whether eNOS in adipocytes could suppress lipolysis in vivo, we administered normal chow (NC) or HFD for 12 weeks to eNOS^-/-^ mice and WT mice. On HFD feeding, eNOS^-/-^ mice showed significant increases in the serum level of FFAs (Fig 3A) as well as in body weight and serum levels of glucose, triglycerides (TG) and cholesterol (S1 Table) compared to WT mice. On HFD, basal serum FFA of eNOS^-/-^ mice was significantly higher than that of WT mice, suggesting that basal lipolysis may be higher in eNOS^-/-^ mice compared to WT mice. Next, to investigate the degree of stimulated lipolysis, isoproterenol was injected interperitoneally and serum FFAs were measured before and 6 hours after the injection. The increase in FFA level was about three times greater in eNOS^-/-^ mice than in WT mice (Fig 3B), indicating that the lack of eNOS in adipocytes also augments stimulated-lipolysis. In support of this result is the observation that the amount of visceral fat was significantly reduced in eNOS^-/-^ mice on HFD compared to their WT counterparts, although subcutaneous fat was increased (Fig 3C). These data suggest that the lack of eNOS in adipocytes augment lipolysis under both basal and stimulated conditions.

We next hypothesized that the excess fatty acids released from visceral fat by lipolysis might accumulate in the liver and thus examined the phenotypic changes of the liver in eNOS^-/-^ mice. Upon HFD feeding, WT mice showed a slight elevation in serum ALT level and liver TG content, but no significant change in liver weight (Fig 3D–3F). In striking contrast, eNOS^-/-^ mice on HFD showed an enlarged liver with dramatically elevated serum ALT and liver TG levels (Fig 3D–3G). In addition, histopathological examination revealed that while WT mice on HFD showed only slight lipid accumulation in the liver, which is consistent with previous reports [12–14], eNOS^-/-^ mice on HFD developed severe macrosteatosis and hepatocyte ballooning (Fig 3H) that were indicative of the presence of nonalcoholic fatty liver disease (NAFLD).

### eNOS^-/-^ Mice Showed Development of NASH

**Fig 4:**
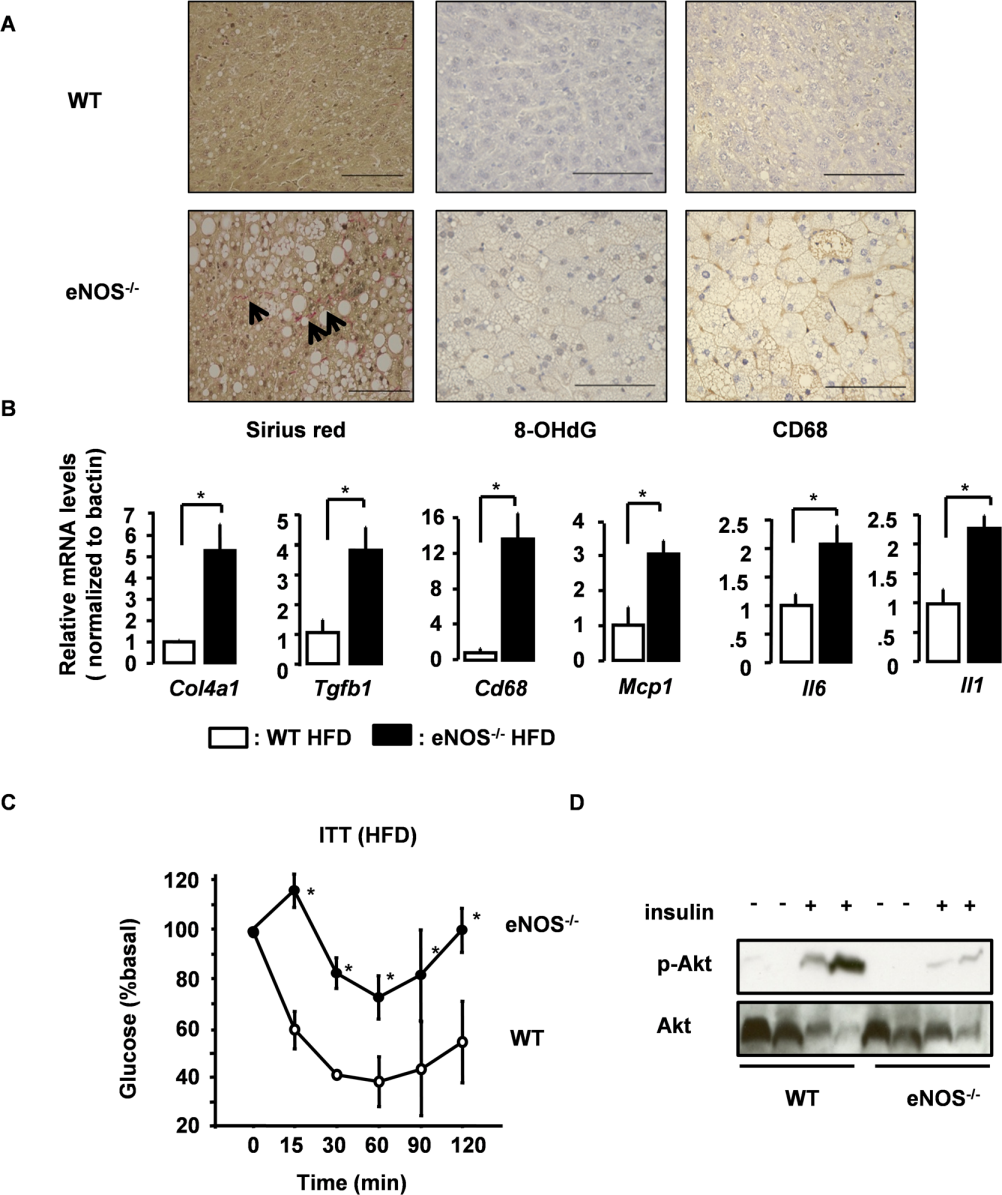
eNOS in adipocytes is critical for preventing NASH development. (A) Livers of HFD-fed WT and eNOS^-/-^ mice were stained with Sirius red, 8-OHdG, or CD68. Arrows indicate areas of fibrosis stained by Sirius red. Scale bar, 100 μm. (B) Expression of fibrosis-related or inflammatory genes in the livers of HFD-fed WT and eNOS^-/-^ mice that were sacrificed in a non-fasted state. Expressions were measured by quantitative real-time RT-PCR assays and normalized to β-actin expression in each sample (mean ± SE; n = 3–4). Statistically significant differences between WT and eNOS^-/-^ are indicated (*p < 0.05). (Tgfb1, transforming growth factorβ1; Col4a1, collagen type IVα1; Mcp1, monocyte chemoattractant protein 1; Il6, interleukin 6; Il1, interleukin 1). (C) Results of insulin tolerance test in HFD-fed WT and eNOS^-/-^ mice (n = 3–5). (D) HFD-fed WT and eNOS^-/-^ mice were fasted overnight and administered insulin (5U) by intravenous injection. Activation of Akt in the liver was examined by immunoblot analysis.

The marked lipid changes seen in eNOS^-/-^ mice on HFD suggest that what was observed in the liver might be more than just simple steatosis. The histopathological findings that are generally required for the diagnosis of NASH (NAFLD activity score) are macrosteatosis, hepatocyte ballooning, and lobular inflammation and fibrosis [15]. As shown in Fig 4A and 4B, all four diagnostic features of NASH, fibrosis, oxidative stress, inflammatory cell accumulation, as well as an associated up-regulation in the expression of fibrosis- and inflammation-related genes, were observed in eNOS^-/-^ mice on HFD. These findings are all suggestive of the development of steatohepatitis rather than simple fatty liver. The oleic acid to stearic acid ratio (C18:1 / C18:0) in the liver was significantly higher in eNOS^-/-^ mice than in WT mice (S3A Fig), which further supports the likely progression from simple fatty liver to hepatitis or liver cirrhosis in eNOS^-/-^ mice [16, 17]. In addition, the intrahepatic composition of omega-3 polyunsaturated fatty acids, especially α-linolenic acid (C18:3) and docosahexaenoic acid (C22:6), was decreased in eNOS^-/-^ mice (S3B Fig). These results indicate that the deletion of the eNOS gene alone is sufficient for inducing NASH development under HFD conditions.

Furthermore, a markedly higher serum level of insulin was also observed in eNOS^-/-^ mice on HFD (S3C Fig). eNOS^-/-^ mice on HFD showed an attenuated response in the insulin tolerance test (ITT) (Fig 4C) but showed glucose tolerance similar to that of WT mice (data not shown), probably owing to a compensatory increase in insulin secretion. This systemic insulin resistance might be associated with the insulin resistance in the liver caused by NASH.

Three putative pathological mechanisms are implicated in the pathogenesis of NASH, namely an increase in fatty acid synthesis, decrease in fatty acid β-oxidation and excess inflow of fatty acids from visceral fat [18]. The mRNA expression levels of SCD1 and FAS, which mediate fatty acid synthesis, were significantly higher in the livers of eNOS^-/-^ mice than in those of WT mice (S3D Fig). On the other hand, there was no significant difference in the mRNA levels of the fatty acid β-oxidation markers CPT1 and PPARα (S3D Fig). Thus, we believe that the development of NASH in eNOS^-/-^ mice might be due to the increase in hepatic de novo lipogenesis as a result of the excess lipolysis in adipose tissue.

### PPARγ Negatively Regulates eNOS Expression in Adipocytes

**Fig 5:**
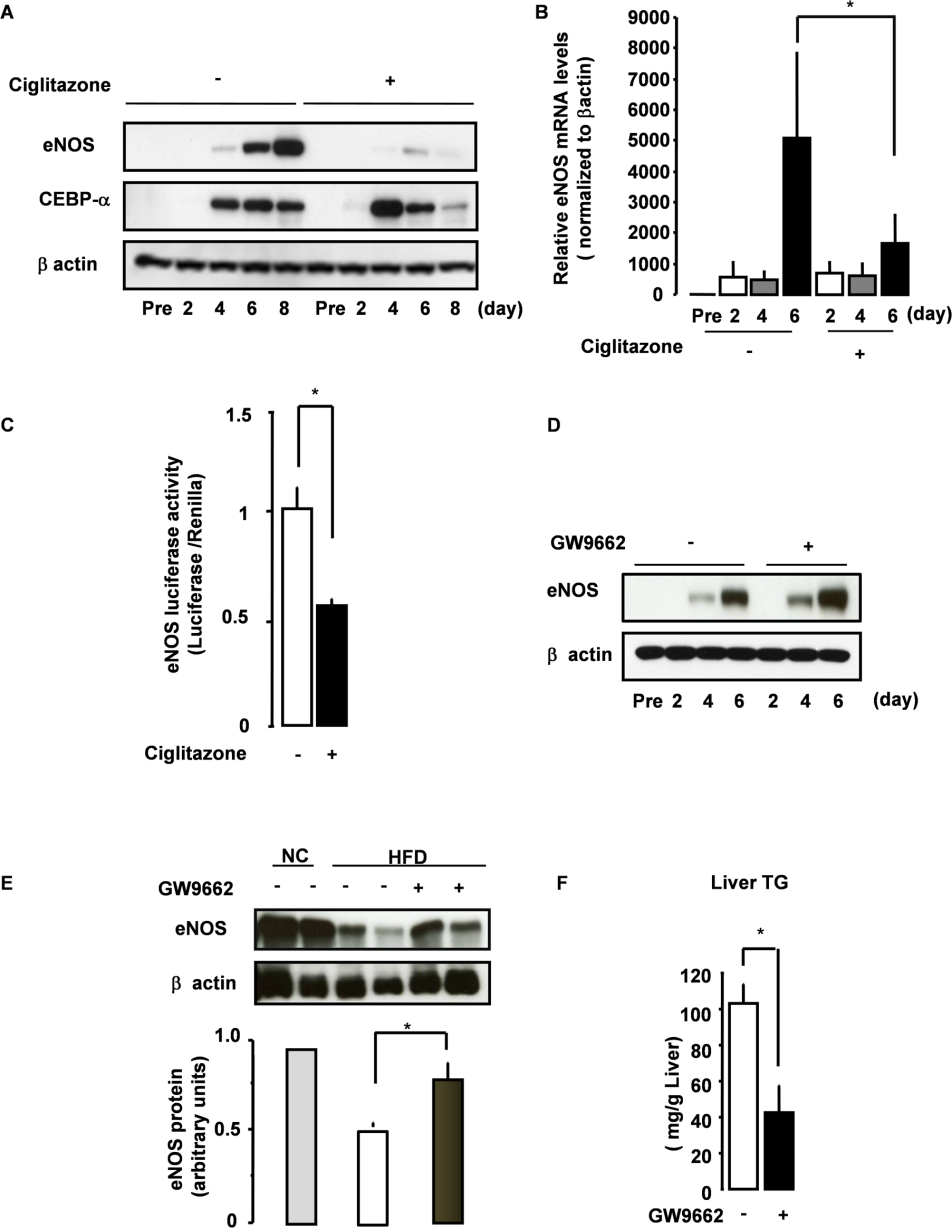
Negative regulatory roles of PPARγ in adipocyte eNOS expression. (A-B) 3T3L1 preadipocytes were pretreated with vehicle or ciglitazone. (A) Cell lysates were analyzed by immunoblot analysis. (B) RNAs were analyzed for eNOS (*Enos*) gene expression by quantitative real-time RT-PCR (n = 4). (C) 3T3L1 adipocytes were pretreated with vehicle or GW9662 (0.01 μM). Cell lysates were then analyzed by immunoblot analysis. (D) 3T3L1 preadipocytes were pretreated with vehicle or ciglitazone (10 μM) with insulin, dexamethasone and IBMX for 48 hours, and then the eNOS-luc construct was transfected. After a further 24 hours, luciferase activity was assayed. Data represent mean ±SEM (n = 6). Statistically significant differences are indicated (*p < 0.05). (E, F) Eight-week-old WT mice on HFD were administered vehicle or GW9662 intraperitoneally (n = 3–5). (E) eNOS expression in epididymal fat was examined by immunoblot analysis. The relative amount of eNOS is presented. (F) Liver TG was measured in HFD-fed, vehicle- or GW9662-treated mice. *p < 0.05. All values are expressed as mean ±SEM.

We next focused on the molecular mechanisms underlying the regulation of eNOS expression in adipocytes. Because eNOS expression was up-regulated during adipocyte differentiation, we focused on PPARγ, the master regulator of adipocyte differentiation [19]. Ciglitazone, a PPARγ agonist, promoted adipocyte differentiation and reduced the size of lipid droplets in adipocytes (Fig 4A). To our surprise, pretreatment of 3T3L1 adipocytes with PPARγ agonists such as ciglitazone and troglitazone either abolished or markedly attenuated the induction of eNOS expression (Fig 5A and 5B and S4B Fig). We also found that ciglitazone significantly reduced eNOS promoter activity (Fig 5C). Consistent with these findings, eNOS expression was increased by pretreatment with the PPARγ antagonist GW9662 (Fig 5D). Taken together, these data suggest a negative regulatory role of PPARγ in adipocyte eNOS expression.

Finally, to examine the regulatory role of PPARγ in adipose eNOS expression in vivo, we administered GW9662 to WT mice on HFD. While there was no difference in food intake between vehicle- and GW9662-treated mice (not shown), treatment with GW9662 prevented the time-dependent increase in weight on HFD (S4C Fig). As shown in Fig 5E, HFD significantly down-regulated eNOS expression in the adipose tissues of vehicle-treated mice while it was preserved in the adipose tissues of mice treated with GW9662. Consistent with this preserved eNOS expression in adipose tissue, the HFD-induced excess liver TG deposition was also prevented by GW9662 (Fig 5F). Accordingly, we observed an inverse relationship between the adipose eNOS level and liver TG content, suggesting that preserving adipocyte eNOS expression with the use of GW9662 may contribute to the mechanism of its prevention of HFD-induced fatty liver disease.

## Discussion

The present study demonstrated that the lack of heterotopic expression of eNOS in adipocytes cause excess lipolysis and contributes to excessive fat storage in the liver and subsequent NASH formation.

It has already been shown that eNOS is expressed in human, mouse and rat adipose tissues, although the regulatory mechanisms and functions of heterotopic eNOS in adipocytes remain unclear [20]. We found that heterotopic expression of eNOS in adipocytes has a suppressive effect on lipolysis. In general, there are two main signaling pathways for the cellular effects of NO. One is via the activation of sGC while the other is through the post-translational modification (S-nitrosylation) of cysteine thiol to form nitrosothiol (SNO) [10, 11]. S-nitrosylation is intimately involved in cellular signal transduction, transcription factor control and apoptosis. Furthermore, S-nitrosylation plays an important role in a broad spectrum of human diseases such as diabetes and cardiovascular disease [21, 22]. In this study, auranofin and DNCB suppressed lipolysis whereas ODQ and vardenafil had no effect, indicating that protein S-nitrosylation is involved in the suppressive effect of eNOS/NO on adipocyte lipolysis. Although the addition of isoproterenol promoted S-nitrosylation in cells, the target protein for S-nitrosylation was not identified in the present study. As eNOS knock-down had no effect on the phosphorylation of HSL (S2F Fig), another lipase or other downstream molecules such as ATGL and perilipin might be the target of S-nitrosylation.

In this study, we observed the development of NASH in HFD-fed eNOS^-/-^ mice, a novel finding that adds to previous reports showing that these mice exhibit the typical features of the metabolic syndrome such as weight gain, insulin resistance, and hypertension [5]. These metabolic syndrome-like phenotypes are mediated by reduced mitochondrial number and reduced energy expenditure [6]. In terms of fatty liver disease development, there are three major possible pathological mechanisms: increased hepatic de novo lipogenesis, decreased β-oxidation in the liver, and increased inflow of FFAs released from adipose tissue to the liver as a result of adipocyte lipolysis. Our data show that NASH formation observed in HFD-fed eNOS^-/-^ mice mainly results from the excess inflow of FFAs into the liver due to increased lipolysis caused by the lack of adipose eNOS expression. The etiology of NASH is considered to be associated with a multiple-hit process involving insulin resistance, adipokines, oxidative stress, and apoptosis [23]. Although several animal models of fatty liver disease such as ob/ob mice, db/db mice, and Zucker rats have been developed, these models show only local liver changes or changes without liver fibrosis [12–14], and most of these models require another stress factor such as a methionine-choline-deficient diet in order for NASH to develop [24]. Indeed, eNOS^-/-^ mice showed a marked progression to NASH upon administration of only HFD, as well as exhibiting features also seen in patients with NASH such as inflammation, liver fibrosis, and systemic insulin resistance. Thus, the suppressive effect on lipolysis of heterotopic eNOS in adipocytes has an important role in the development of NASH, and it might be important for the prevention of NASH to maintain or even up-regulate the expression of eNOS in adipocytes.

We found that the expression of eNOS in adipocytes was induced during adipocyte differentiation in vitro and decreased by HFD administration in vivo. Furthermore, PPARγ, which is known to be the master regulator of adipocyte differentiation [19], had a negative regulatory role in adipocyte eNOS expression, although the direct interaction of eNOS and PPARγ remains undetermined. Agonist-induced activation of PPARγ has been demonstrated to increase insulin sensitivity [25, 26] and thiazolidinediones (TZD) are used clinically to reduce insulin resistance and hyperglycemia in patients with type 2 diabetes, although these drugs are also associated with weight gain [27–31]. It has also been reported that adipocyte-specific overexpression of PPARγ induced adipogenic steatosis in the mouse liver [32]. On the other hand, the moderate reduction of PPARγ activity observed in heterozygous PPARγ-deficient mice and with the use of PPARγ antagonists has been reported to prevent fatty liver disease induced by HFD [33–36]. Following these reports, we also observed that administration of a PPARγ antagonist to HFD-fed mice diminished HFD-induced obesity and fatty liver disease while being associated with the restoration of eNOS expression in adipocytes. Given the inverse relationship between adipose eNOS expression and hepatic TG content, it is possible that restoration of adipose eNOS expression and the subsequent suppression of adipose tissue lipolysis might also contribute to how a PPARγ antagonist prevents HFD-induced fatty liver disease (S5 Fig).

In conclusion, adipose tissue-specific eNOS has a suppressive action on lipolysis and contributes to the prevention of NASH development, and up-regulation of adipose eNOS expression by a PPARγ antagonist may present a new therapeutic approach to the treatment of NASH and other related atherosclerotic cardiovascular diseases.

## Supporting Information

S1 Fig Expression of eNOS in adipocytes. (TIF)

S2 Fig Effect of adipocyte-specific eNOS. (TIF)

S3 Fig NASH in eNOS-/- mice on HFD. (TIF)

S4 Fig Roles of PPARγ in adipocyte eNOS expression. (TIF)

S5 Fig Suppressive role of adipocyte-expressing eNOS in lipolysis. (TIF)

S1 File Supporting figure legends and Supplementary methods. (DOC)

S1 Table Phenotypic characteristics of wild type (WT) and eNOS-/- mice fed normal chow or HFD. (DOC)
