# Supplementary figures and images for: Suppressive Role of PPARγ-Regulated Endothelial Nitric Oxide Synthase in Adipocyte Lipolysis
Source: PLoS One. 2015 Aug 28;10(8):e0136597. doi: 10.1371/journal.pone.0136597 (PMC4552558; doi:10.1371/journal.pone.0136597)

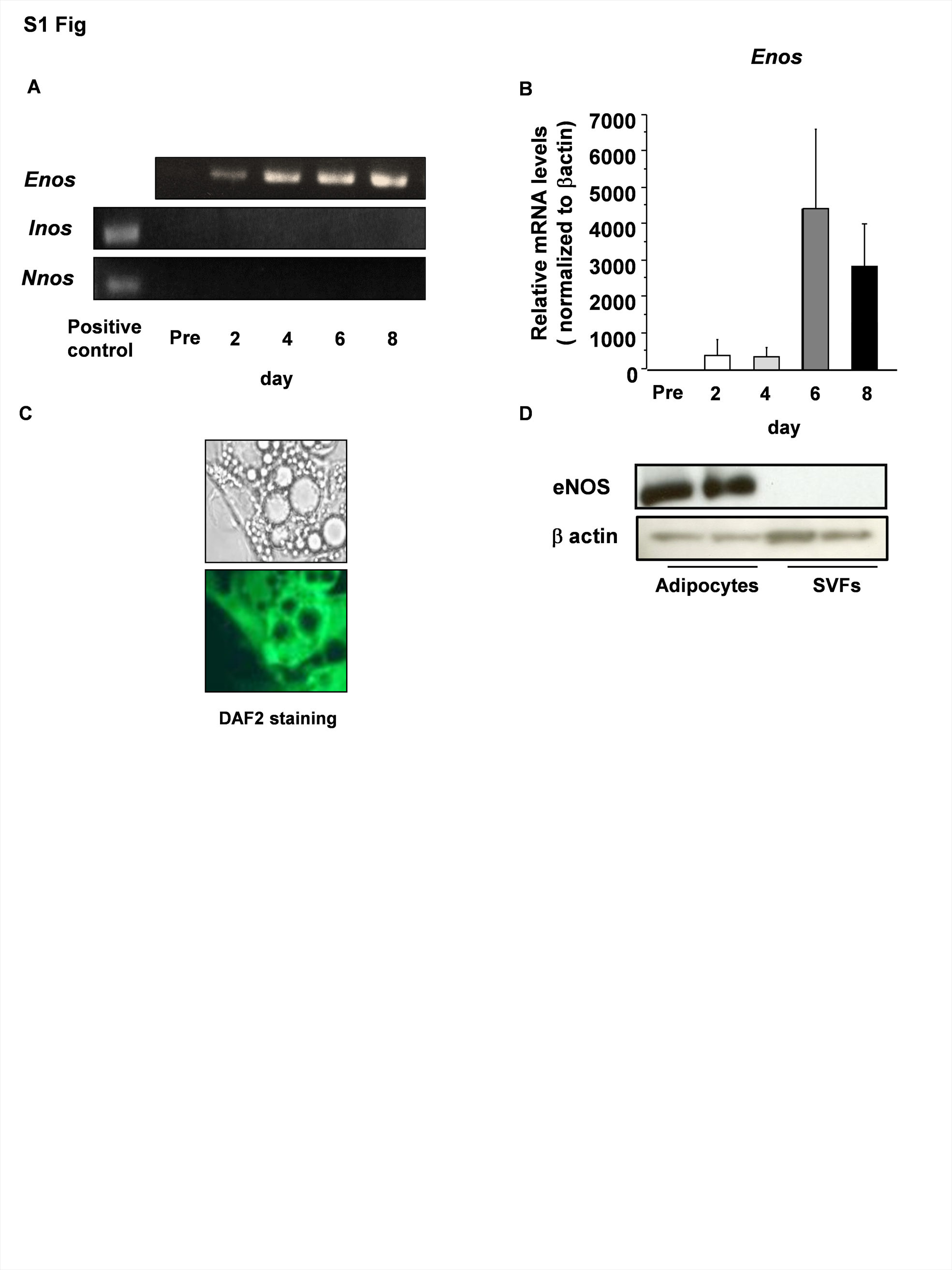

Supplement: S1 Fig — (TIF) [file pone.0136597.s001.tif]

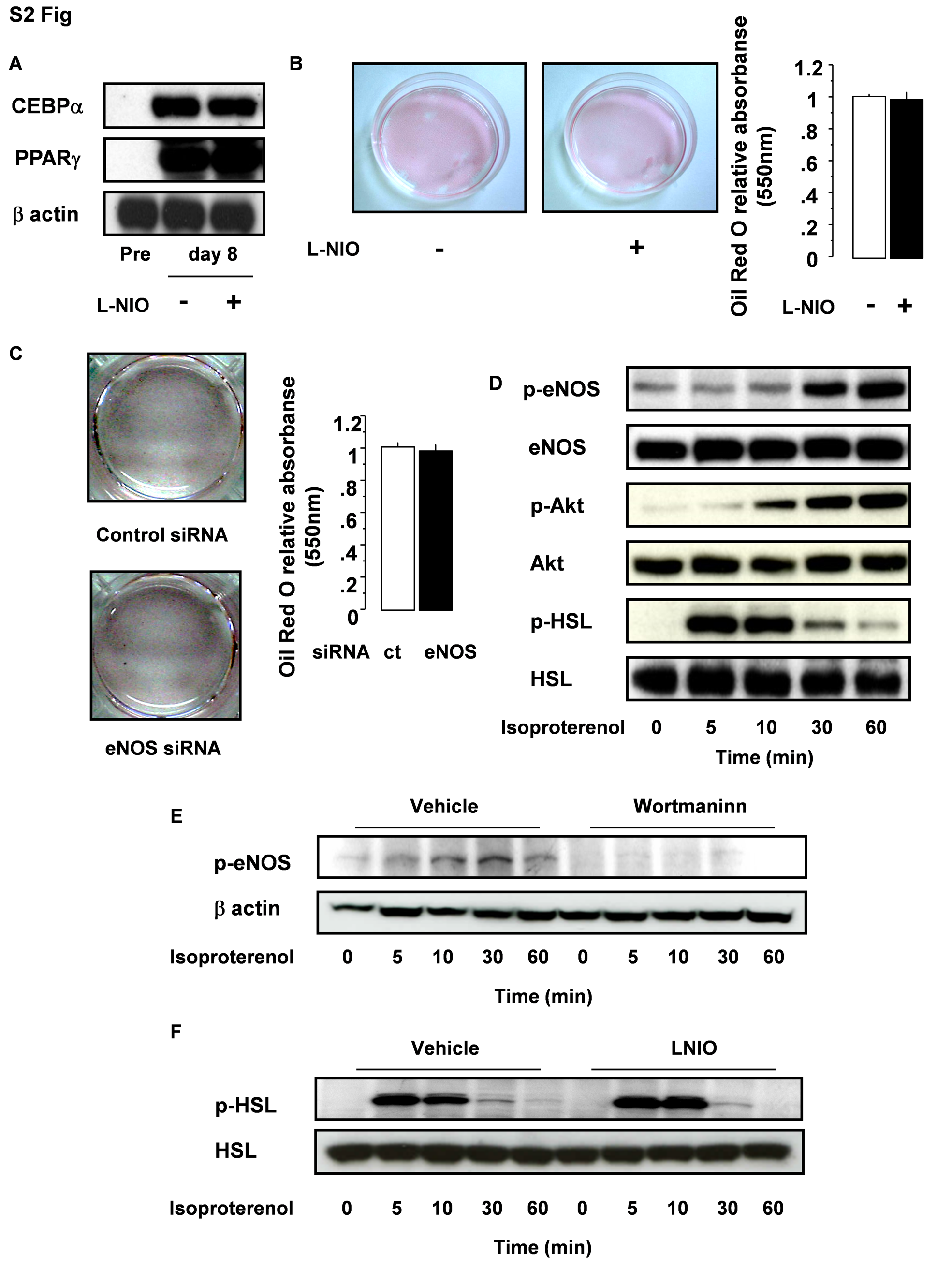

Supplement: S2 Fig — (TIF) [file pone.0136597.s002.tif]

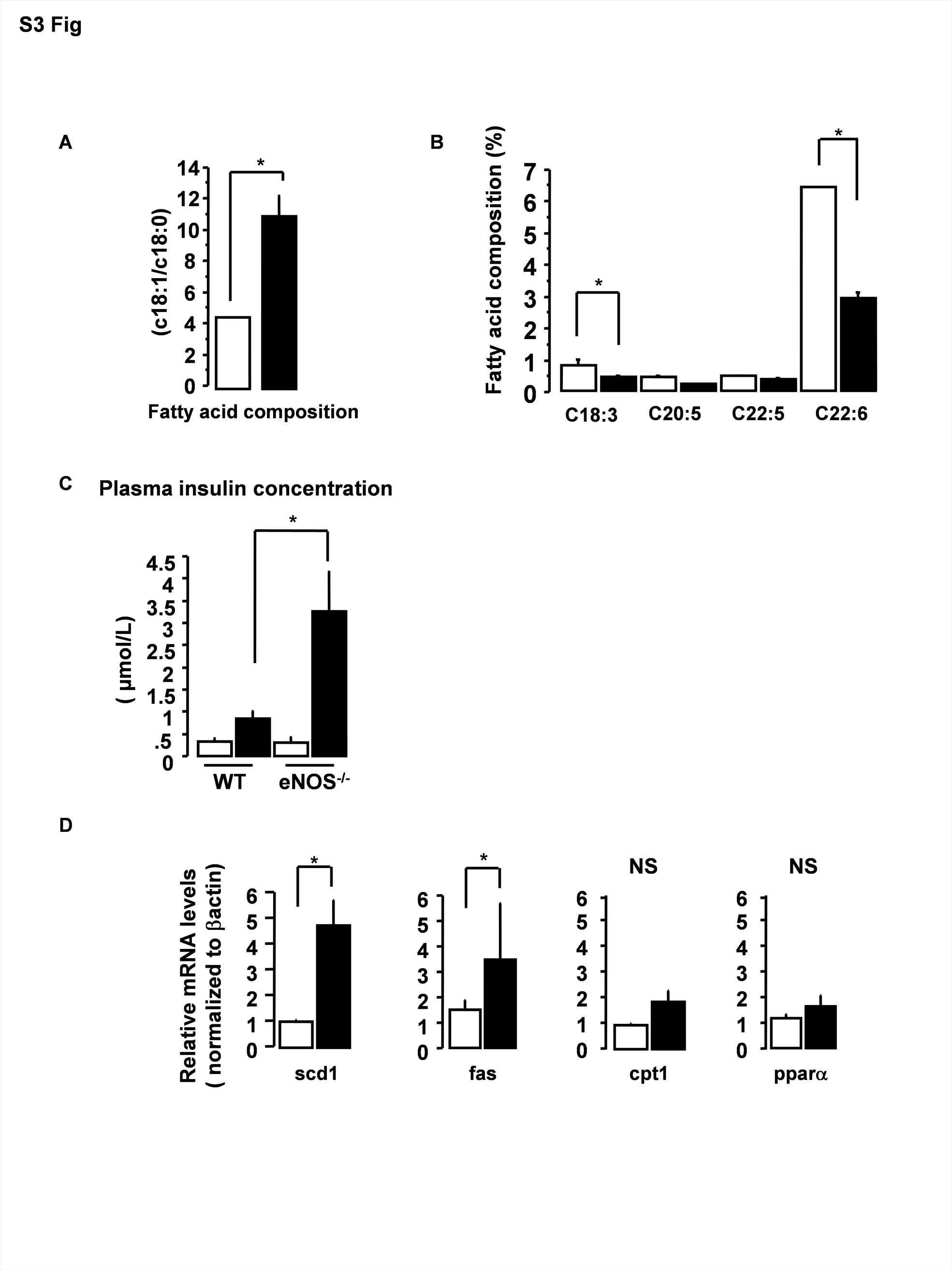

Supplement: S3 Fig — (TIF) [file pone.0136597.s003.tif]

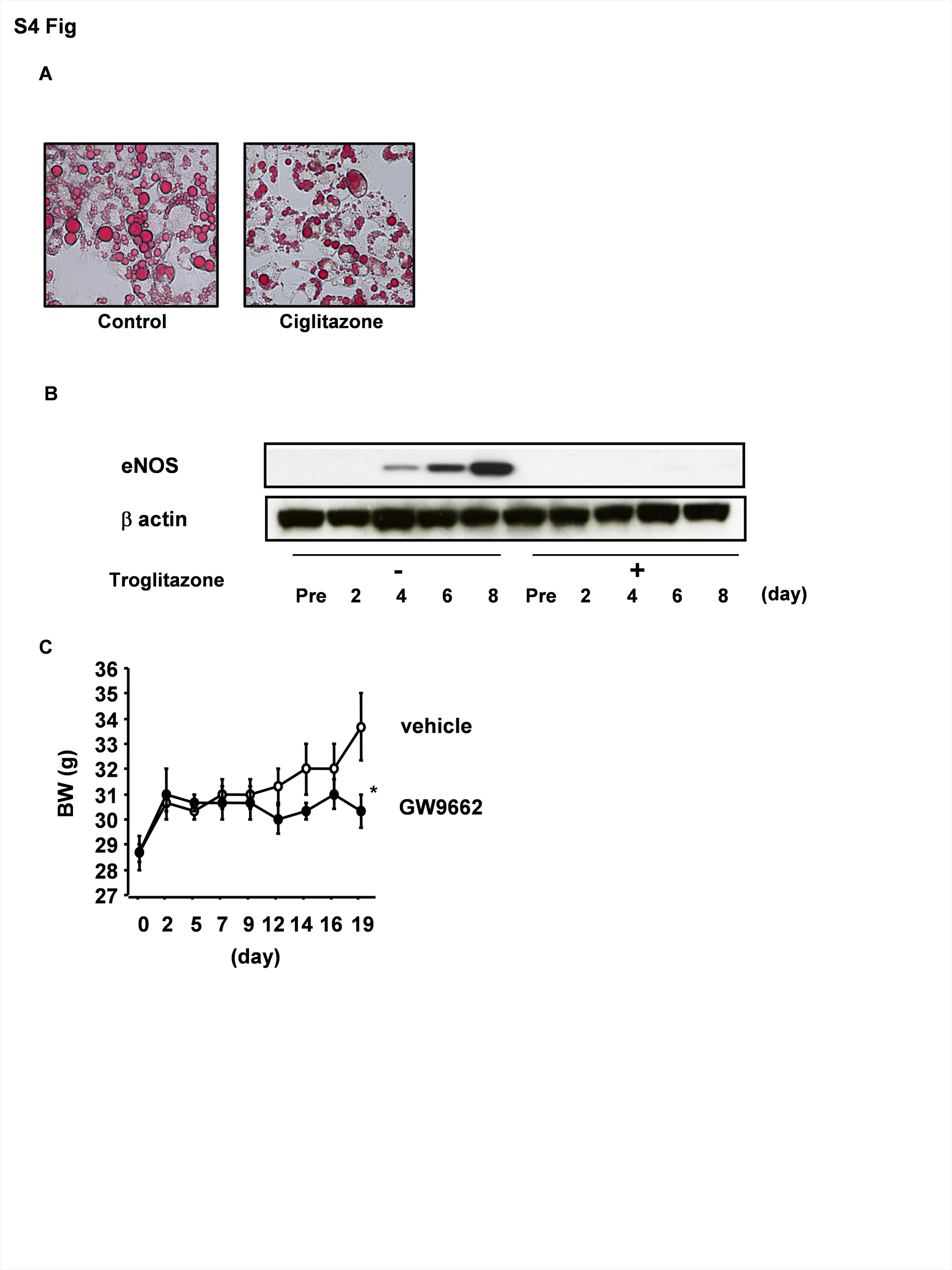

Supplement: S4 Fig — (TIF) [file pone.0136597.s004.tif]

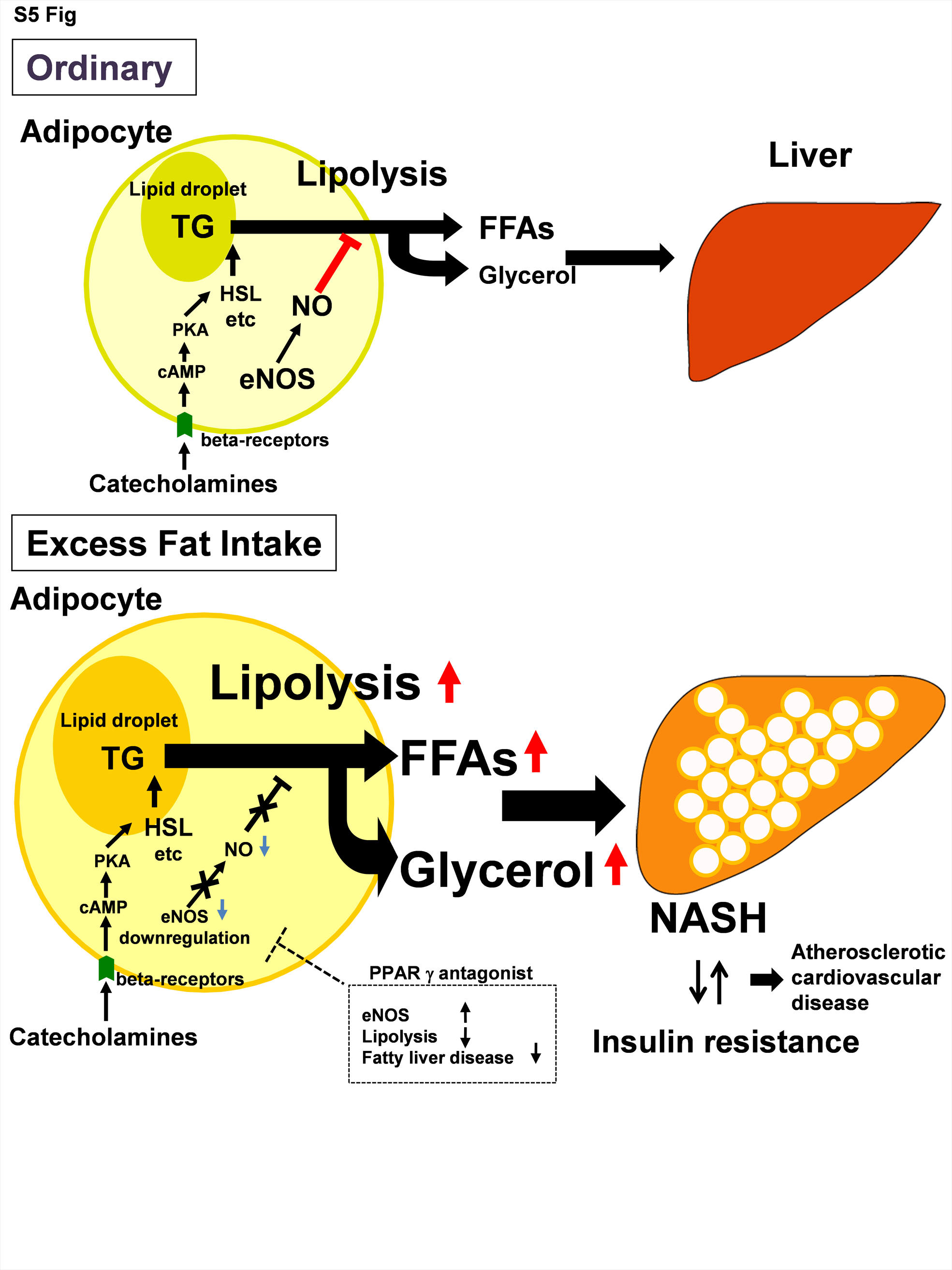

Supplement: S5 Fig — (TIF) [file pone.0136597.s005.tif]
