# Supplementary material for: Suppressive Role of PPARγ-Regulated Endothelial Nitric Oxide Synthase in Adipocyte Lipolysis
Source: PLoS One. 2015 Aug 28;10(8):e0136597. doi: 10.1371/journal.pone.0136597 (PMC4552558; doi:10.1371/journal.pone.0136597)
Supplement: S1 File — (DOC) [file pone.0136597.s006.doc]

**Supplementary Figure Legends**

**S1 Fig.**

(A) RT-PCR analysis of eNOS (Enos), iNOS (Inos) and nNOS (Nnos) gene expression during 3T3L1 adipocyte differentiation. (B) Quantitative RT-PCR analysis of eNOS (Enos) gene expression during 3T3L1 adipocyte differentiation. (C) Microscopic images of 3T3L1 adipocytes without staining (upper) and with DAF2 staining (lower). Photos were taken under a microscope with a ×40 objective lens. (D) Extracts prepared from adipocytes and stromal-vascular fractions (SVF) derived from epididymal fat of wild type mice were examined by immunoblot analysis by probing with antibody to eNOS.

**S2 Fig.**

(A-B) 3T3L1 adipocytes were pretreated with vehicle or L-NIO (100 mM) from the day before adding insulin, dexamethasone and IBMX to day 8 on alternate days. (A) Cell lysates were then analyzed by immunoblot analysis by probing with antibodies to C/EBPα and PPARγ. (B) Oil red O staining on day 8 and relative quantification of adipocyte differentiation. (C) 3T3L1 adipocytes were pretreated with control siRNA or eNOS-siRNA (200 pM) for every 48 hours from the day before adding insulin, dexamethasone and IBMX until day 6. Oil red O staining on day 8 and relative quantification of adipocyte differentiation. (D) Lipolysis was induced by addition of isoproterenol (10 mM) to mature 3T3L1 adipocytes for 1 hour. Cell lysates were collected and then analyzed by immunoblot analysis by probing with antibodies to Ser-1177-phosphorylated eNOS, total eNOS, Ser-473-phosphorylated Akt, total Akt, Ser-660-phosphorylated HSL and total HSL. (E) Mature 3T3L1 adipocytes (day 10) were preincubated with vehicle or wortmaninn for 24 hours. Then, lipolysis was induced by the addition of isoproterenol for 1 hour. Cell lysates were collected at 0, 5, 10, 30 and 60 min and then analyzed by immunoblot analysis by probing with antibody to Ser-1177-phosphorylated eNOS. (F) Mature 3T3L1 adipocytes (day 10) were preincubated with vehicle or L-NIO (100 mM) for 24 hours. Then, lipolysis was induced by the addition of isoproterenol for 1 hour. Cell lysates were collected at 0, 5, 10, 30 and 60 min and then analyzed by immunoblot analysis by probing with antibody to Ser-660 of HSL and for total HSL.

**S3 Fig.**

(A) Ratio of oleate (18:1) to stearate (18:0) in livers of HFD-fed WT and eNOS^-/-^ mice. Data represent mean ±SEM. Statistically significant difference is indicated (*p < 0.05). (B) Intrahepatic composition of omega-3 polyunsaturated fatty acids of liver tissue in HFD-fed WT and eNOS-/- mice. Data represent mean ±SEM. Statistically significant differences are indicated (*p < 0.05). (C) Serum insulin level in NC- and HFD-fed WT and eNOS^-/-^ mice (n = 4-8). *p < 0.05. All values are expressed as mean ±SEM. (D) Expression of genes associated with fatty acid metabolism in the livers of HFD-fed wild type (WT) and eNOS^-/-^ mice was measured by quantitative real-time RT-PCR assays and normalized to the amount of b-actin in each sample (n = 3) (*Scd1*; SCD-1, *Fas*; FAS, *Cpt1*; CPT1, carnitine palmitoyltransferase-1; *Ppara*; PPARα).

**S4 Fig.**

(A) Oil red O staining of 3T3L1 adipocytes on day 10. 3T3L1 preadipocytes were treated with vehicle or ciglitazone (10 mM). (B) 3T3L1 preadipocytes were pretreated with vehicle or troglitazone (10 mM) from the day before adding insulin, dexamethasone and IBMX to day 8 on alternate days. Cell lysates were then analyzed by immunoblot analysis by probing with antibody to eNOS. (C) 8-week-old-male WT mice were treated with vehicle or GW9662 (10mg/kg) interperitoneally on alternate day for 19 days on HFD (n = 3). Body weight values of HFD-fed vehicle- and GW9662-treated mice. *p < 0.05. All values are expressed as mean ±SEM.

**S5 Fig.**

Suppressive role of adipocyte-expressing eNOS in lipolysis

In normal condition, adipocytes express eNOS, which has a suppressive effect on lipolysis to prevent excess FFA release from adipocytes. HFD induces a decrease in adipocyte eNOS expression, which could lead to augmented lipolysis, excess inflow of FFAs to the liver and NASH formation. This pathological pathway could be prevented by a PPARγ antagonist via restoration of the eNOS downregulation induced by HFD.

**Supplementary Methods**

**Fatty acid composition in liver**

An aliquot (0.1 g) of liver sample snap-frozen by liquid nitrogen was homogenized in 1 mL normal saline. The fatty acid composition was measured by gas chromatography at Bio-Medical Laboratories. Briefly, total lipids in liver homogenates were extracted according to Folch’s procedure , followed by transesterification of fatty acids with boron trifluoride-methanol at 100°C for 90 minutes. The methylated fatty acids were then extracted with hexane and analyzed using a GC-17A gas chromatograph (Shimadzu Corporation) and BPX70 capillary column (0.25 mm ID × 30 m, SGE International Ltd., Melbourne, Australia).

**Oil red O staining**

Oil red O stock solution (0.5%) was prepared in 60% triethyl phosphate and filtered in cellulose nitrate filters as described previously . The stock solution was diluted 6:4 in water and double filtered before use. Cells were washed 3 times with PBS and then fixed with a fixing solution (4% paraformaldehyde-0.1 M sodium phosphate, pH 7.3) for 30 min before staining for 1 h with Oil Red O working solution, and then washed with tap water. The Oil red O retained in the cells was extracted with isopropanol and quantified by measuring absorbance at 550nm.

**Immunoblotting**

Cells and tissue samples were lysed on ice for 1 hour in buffer (50 mmol/L Tris-HCl, pH 7.6, 150 mmol/l NaCl, 1% NP-40, 0.1% sodium dodecyl sulfate (SDS), 1 mmol/L dithiothreitol, 1 mmol/L sodium vanadate, 1 mmol/L phenylmethylsulfonyl fluoride, 10 µg/mL aprotinin, 10 µg/mL leupeptin, and 10 mmol/L sodium fluoride). Equal amounts of protein were separated by SDS-polyacrylamide gel electrophoresis and transferred to nitrocellulose membranes. After blocking, the filters were incubated with the following antibodies; anti-eNOS (610296; BD Transduction Laboratories), anti-HSL, p-HSL, p-eNOS, Akt, p-Akt, CEBPα, CEBPβ (4107, 4139, 9570, 4691, 9271, 4843, 3087; Cell Signaling), anti-PPARγ(7196; Santa Cruz Biotechnology Inc.), and anti-β-actin (1305567; Sigma). After washing and incubation with horseradish peroxidase-conjugated antirabbit or antimouse immunoglobulin G (Amersham) for 1 hour, antigen-antibody complexes were visualized using an enhanced chemiluminescence system (Amersham).

**Real-time quantitative reverse transcription**

Total RNA in cells and tissue samples were isolated with ISOGEN (Nippon Gene Inc) or an RNeasy Lipid Tissue Mini Kit (QIAGEN). After treatment with Rnase-free Dnase for 30 minutes, total RNA (50 ng/μL) was reverse transcribed with random hexamers and oligo d (T) primers. The expression level of each transcript was determined by means of staining with SYBR green dye and a LineGene fluorescent quantitative detection system (Bioflux Co), as recommended by the manufacturer. Primer quality was verified by dissociation curve analysis, the slopes of standard curves, and reactions without RT.

The primer sets were as follows;

*eNOS* forward, 5’- TTCCGGCTGCCACCTGATCCTAA -3’,

*eNOS* reverse, 5’- AACATATGTCCTTGCTCAAGGCA -3’;

*Scd1* forward, 5’- CGGCGCGGAAGCTGT-3’,

*Scd1* reverse, 5’- TGCAATCCATGGCTCCGT-3’;

*Fas* forward, 5’- CCTCAGGGTACCACTACGGAGT-3’,

*Fas* reverse, 5’- GCCGAATAGTTCGCCGAA-3’,

*Cpt1* forward, 5’- CCTGAAGTGCTCGACATCACA-3’,

*Cpt1* reverse, 5’- GCGCTTGTACCCATTGATGA-3’.

*Ppara* forward, 5’- AGAGCCCCATCTGTCCTCTC -3

*Ppara* reverse, 5’- ACTGGTAGTCTGCAAAACCAAA -3

*Mcp1* forward, 5’- CCACTCACCTGCTGCTACTCA -3

*Mcp1* reverse, 5’- TGGTGATCCTCTTGTAGCTCTCC -3

*Col4a1* forward, 5’- CTGGCACAAAAGGGACGAG -3

*Col4a1* reverse, 5’- ACGTGGCCGAGAATTTCACC -3

*Tgfb1* forward, 5’- CTCCCGTGGCTTCTAGTGC -3

*Tgfb1* reverse, 5’- GCCTTAGTTTGGACAGGATCTG -3

*Cd68* forward, 5’- GGACCCACAACTGTCACTCAT -3

*Cd68* reverse, 5’- AAGCCCCACTTTAGCTTTACC -3

*Il6* forward, 5’- TAGTCCTTCCTACCCCAATTTCC -3

*Il6* reverse, 5’- TTGGTCCTTAGCCACTCCTTC -3

*Il1* forward, 5’- GCAACTGTTCCTGAACTCAACT -3

*Il1* reverse, 5’- ATCTTTTGGGGTCCGTCAACT -3

**Preparation of small interfering RNA targeting**

Twenty-four hours after seeding of 3T3L1 pre-adipocytes onto 6-well plates or 6 days after the addition of differentiation cocktail, cells were transfected with 200 pM siRNA for eNOS AAA UUA AUG UGG CCG UGU UUU and AAC ACG GCC ACA UUA AUU UUU (Dharmacon ON-TARGET plus SMART pool siRNA) and control siRNA using silMPORTER (Upstate) every time the medium was changed (every 2 days) up to 8 days. The loss of eNOS by transfection of siRNA was validated by immunoblotting for eNOS protein in the cell lysates 48 hours after siRNA transfection.

**REFERENCES**

1. Folch J, Lees M, Sloane Stanley GH. A simple method for the isolation and purification of total lipides from animal tissues. J Biol Chem. 1957;226(1):497-509. Epub 1957/05/01. PubMed PMID: 13428781.

2. Koopman R, Schaart G, Hesselink MK. Optimisation of oil red O staining permits combination with immunofluorescence and automated quantification of lipids. Histochem Cell Biol. 2001;116(1):63-8. Epub 2001/08/02. PubMed PMID: 11479724.
