# Supplementary material for: Suppressive Role of PPARγ-Regulated Endothelial Nitric Oxide Synthase in Adipocyte Lipolysis
Source: PLoS One. 2015 Aug 28;10(8):e0136597. doi: 10.1371/journal.pone.0136597 (PMC4552558; doi:10.1371/journal.pone.0136597)
Supplement: S1 Table — (DOC) [file pone.0136597.s007.doc]

**S1 Table: Phenotypic characteristics of wild type (WT) and eNOS^-/-^** mice fed normal chow or HFD.

|  | **WT**  **Normal chow** | **WT**  **HFD** | **eNOS ^-/-^**  **Normal chow** | **eNOS ^-/-^**  **HFD** |
| --- | --- | --- | --- | --- |
| **Body weight**  **(g)** | **29.92±1.74** | **37.06±3.41*** | **25.90±2.23** | **41.22±4.31 ^✝^, **** |
| **Glucose**  **(mg/dL)** | **101.5±20.0** | **232.3±58.7*** | **246.6±148.0*** | **272.0±166.8 ^✝^, **** |
| **Triglycerides**  **(mg/dL)** | **30.2±5.4** | **50.3±10.7** | **32.7±17.2** | **80.5±45.4 ^✝^, **** |
| **Cholesterol**  **(mg/dL)** | **81.1±9.1** | **152.1±27.8*** | **76.0±13.4** | **209.6±33.3 ^✝^, **** |

Values are mean ±SEM. for n = 5-7 mice.

***** : vs WT (normal chow) ,^✝^: vs WT (HFD), **: vs eNOS^-/-^ (normal chow)

Asterisks represent significant difference of at least p < 0.05.
